# Longitudinal study of factors associated with the anti-cancer efficacy and liver function in HCC patients treated with TACE in combination with percutaneous ablation

**DOI:** 10.3389/fonc.2025.1566865

**Published:** 2025-04-16

**Authors:** Huhu Ren, Jian Chen, Zhiqun Wu, Chen Li

**Affiliations:** Hong Hui Hospital, Xi’an Jiaotong University, Xi’an, China

**Keywords:** hepatocellular carcinoma, TACE, percutaneous ablation, treatment efficacy, prognostic factors

## Abstract

**Background:**

Hepatocellular carcinoma (HCC) is a major cancer challenge worldwide. Combination therapy using transcatheter arterial chemoembolization (TACE) and percutaneous ablation offers potential for improved outcomes.

**Objective:**

To evaluate the efficacy and liver function preservation in HCC patients treated with combined TACE and percutaneous ablation, identifying key prognostic factors.

**Methods:**

This longitudinal study included 200 HCC patients. Factors analyzed included tumor characteristics, liver function tests, and serologic markers. Statistical analyses determined associations with treatment outcomes and survival.

**Results:**

Smaller tumors (≤5.0 cm) and lower AFP levels (<200 ng/mL) were associated with higher treatment efficacy, with an objective response rate of 67.3% for lower AFP levels versus 42.3% for higher levels. Liver function was better preserved in patients with lower AFP levels (78.2% vs. 57.7%). Tumor size and liver stiffness significantly influenced survival and liver function outcomes.

**Conclusion:**

The combination of TACE and percutaneous ablation enhances outcomes in HCC, guided by specific prognostic markers. This supports the need for personalized approaches in HCC treatment and further research into combination therapies.

## Introduction

Hepatocellular carcinoma (HCC) is the sixth most prevalent cancer and the third leading cause of cancer-related mortality worldwide (1, 2). The incidence of HCC is notably high in east Asia, where hepatitis B virus (HBV) remains a predominant cause (3). The incidence is rising due to metabolic dysfunction-associated steatotic liver disease (MASLD) and alcohol-related liver disease (ALD) in Western countries (4, 5). Globally, HCC accounts for approximately 830,000 deaths annually (3, 6). The etiology of HCC is often multifactorial including chronic infections from HBV and hepatitis C virus (HCV), excessive alcohol consumption, and nonalcoholic fatty liver disease (NAFLD) (1, 7). Clinically, HCC often presents asymptomatically in its early stages, leading to delayed diagnoses and poorer prognoses (1).

Treatment strategies for HCC vary based on disease stage and include curative approaches such as surgical resection and liver transplantation (4, 8). Local ablative therapies, including radiofrequency ablation (RFA) and microwave ablation (MWA), are effective for small tumors, while emerging techniques like cryoablation, irreversible electroporation (IRE), and laser ablation offer potential advantages despite limitations such as cryoshock and electrode placement challenges (4). For intermediate-stage disease, loco-regional treatments like transcatheter arterial chemoembolization (TACE) and transarterial radioembolization (TARE) are recommended (2, 4). Systemic therapies, including tyrosine kinase inhibitors like sorafenib and lenvatinib, and immunotherapies such as checkpoint inhibitors (atezolizumab, pembrolizumab), are increasingly utilized, particularly for advanced-stage HCC (4, 8).

TACE and ablation therapies face significant challenges due to resistance mechanisms. One primary mechanism of resistance in TACE is hypoxia-induced angiogenesis, where elevated levels of vascular endothelial growth factor (VEGF) and other growth factors promote new blood vessel formation (9, 10). Tumor heterogeneity also contributes to incomplete necrosis, leading to residual viable tumor cells (11). In ablation therapies, the heat-sink effect, particularly in RFA and MWA, poses a challenge by dissipating heat away from the target area, thus preventing adequate thermal destruction of tumor cells (12). Additionally, achieving an adequate margin in large or irregular lesions is difficult, increasing the risk of residual tumor and early recurrence (11). Clinically, these resistance mechanisms can result in residual viable tumor, intrahepatic spread, or early recurrence, significantly impacting patient outcomes (13).

The overall survival and prognosis of patients are heavily influenced by the stage of the disease, with early-stage patients having a 60–70% 5-year survival rate with curative options, while advanced or metastatic disease sees a significant drop in survival rates (12). Comorbidities and liver function also play crucial roles in determining survival outcomes (12). TACE is associated with post-embolization syndrome and the risk of hepatic decompensation, often necessitating repeat sessions (9). Ablative therapies are limited by maximum lesion size constraints and potential damage to adjacent structures, with risks of hemorrhage and procedure-related complications such as cryoshock in cryoablation (12). Where TACE-induced ischemia can shrink or devitalize tumor tissue, facilitating more complete thermal ablation and potentially extending the size limit for effective ablation (9). Studies suggest that this combination therapy may improve outcomes compared to TACE alone in select patients, enhancing both survival and local control (9).

Identifying factors that predict treatment outcomes in HCC is crucial for optimizing therapeutic strategies and improving patient survival rates. Various studies have highlighted significant clinical, laboratory, and imaging predictors that can guide treatment decisions. Factors such as the absence of extrahepatic metastasis, portal vein thrombosis, and specific blood markers have been associated with better progression-free and overall survival in patients receiving immune checkpoint inhibitors and kinase inhibitors (14). Additionally, advanced imaging techniques, including multiparametric MRI, enhance prognostic accuracy by integrating clinical data with radiomic features (15). Furthermore, nomogram models incorporating clinical characteristics like tumor size and alpha-fetoprotein levels have been developed to predict outcomes in patients undergoing chemotherapy (16). These predictive models are essential for personalizing treatment approaches, ultimately leading to improved management of HCC (17, 18).

The primary objective of this study is to evaluate the efficacy of combining TACE with percutaneous ablation in treating HCC. The research evaluates the impact of this integrated treatment approach on anti-cancer efficacy and liver function preservation in HCC patients. Specifically, we aim to identify predictors for treatment success and contribute to refining therapeutic strategies for HCC.

## Materials and methods

### Study design and ethical approval

This was a retrospective cohort study conducted at Hong hui Hospital, Xi’an Jiaotong University. From January 2015 to December 2018, we enrolled 200 patients diagnosed with hepatocellular carcinoma (HCC) who underwent TACE followed by percutaneous ablation (radiofrequency or microwave). The study protocol was reviewed and approved by the Institutional Review Board of Hong hui Hospital, Xi’an Jiaotong University. Patient confidentiality was strictly maintained in accordance with the Declaration of Helsinki.

*Inclusion Criteria:* 1), Age ≥18 years; 2), HCC diagnosis confirmed by dynamic imaging (CT/MRI) and/or histopathology, according to established guidelines; 3), At least one session of TACE plus percutaneous ablation performed during the study period; 4), Adequate liver function (Child-Pugh class A or B) and an Eastern Cooperative Oncology Group (ECOG) performance status of 0 or 1; 5), Available follow-up data (imaging, laboratory tests) for at least 6 months after the initial therapy or until death.

*Exclusion Criteria:* 1), Previous liver resection or transplantation prior to TACE plus percutaneous ablation; 2), Extrahepatic metastasis at baseline (e.g., lung, bone) or diffuse tumor burden precluding locoregional treatment; 3), Main portal vein thrombosis (macrovascular invasion in the main trunk); 4), Incomplete clinical data or lost to follow-up prior to the first assessment; 5), Child-Pugh class C or active uncontrolled infection.

### Treatment procedures

*TACE Procedure:* All TACE procedures were performed under digital subtraction angiography (DSA) guidance. The femoral artery was accessed using the Seldinger technique, and a catheter was advanced into the hepatic artery. Selective or super-selective catheterization of tumor-feeding arteries was performed whenever feasible. A mixture of chemotherapeutic agent (e.g., doxorubicin, epirubicin) and lipiodol was injected, followed by embolic particles (gelatin sponge particles) to achieve stasis in the segmental/subsegmental arteries. Patients were monitored for post-embolization syndrome (fever, abdominal pain, nausea) and received supportive care (analgesics, antiemetics, intravenous fluids) as needed.

*Percutaneous Ablation:* Percutaneous ablation (either radiofrequency ablation or microwave ablation) was typically performed 1–2 weeks after TACE, once liver enzymes and patient status were optimized. Under ultrasound or CT guidance, an electrode/probe was inserted into the target lesion. For radiofrequency ablation, energy was delivered for the recommended duration until achieving an adequate ablation zone encompassing the tumor and a ~0.5–1 cm margin. For microwave ablation, similar protocols were applied depending on lesion size and location. Vital signs were closely observed to detect complications (e.g., hemorrhage, adjacent organ injury). Follow-up CT or MRI was performed within 4–6 weeks to evaluate the ablation zone.

### Data collection

Baseline demographics (age, sex, etiology of liver disease), clinical profiles (ECOG status, Child-Pugh class, albumin-bilirubin [ALBI] grade, Model for End-Stage Liver Disease [MELD] score), and tumor characteristics (size, number of nodules, macrovascular invasion, alpha-fetoprotein [AFP] level) were extracted from medical records. AFP was recorded at baseline and during follow-up. In some patients, inflammatory markers (NLR, CRP) were collected if routinely measured. Transient elastography (FibroScan^®^) or shear-wave elastography was performed within 3 months before TACE, the stiffness value (kPa) was recorded.

### Risk score calculation and stratification

The hazard ratio (HR) of identified independent prognostic factors via multivariate Cox regression was converted to a point value by comparing its Cox regression coefficient (*β*) to that of the smallest *β* (our reference factor) and rounding to the nearest integer. The high-risk and low-risk score were determined by Kaplan-Meier survival analysis with best stratification of the populations.

### Statistical analysis

All analyses were performed using SPSS version 28. Categorical variables are expressed as frequencies and percentage, while continuous variables are expressed as median (range) or mean ± SD as appropriate. Kaplan–Meier method was used to estimate survival curves for OS and RFS. Differences between groups were compared by the log-rank test. Hazard ratios (HRs) and 95% confidence intervals (CIs) were calculated via univariate Cox regression to identify potential prognostic factors (tumor size, Child-Pugh class, AFP, etc.). Variables with p < 0.05 in univariate testing (or of clinical relevance) were entered into a Cox proportional hazards model to estimate independent predictors of survival. Adjusted HRs (95% CI) and p-values were reported. A point-based risk score was derived, patients were stratified into low- vs. high-risk (or multi-tier) groups based on a chosen cutoff. Survival curves and log-rank p-values were then compared among these subgroups. A two-sided p < 0.05 was considered statistically significant.

## Result

### Patient and treatment characteristics in HCC therapy

A total of 200 HCC patients treated with TACE combined with percutaneous ablation were enrolled in this study. The baseline clinical characteristics were presented in **Table 1**.

**Table 1:** Clinical characteristics of participants.

Characteristic	N = 200
Age (years), median (range)	59 (34–78)
Sex	
Male	161 (80.5%)
Female	39 (19.5%)
Etiology of Liver Disease	
Hepatitis B	125 (62.5%)
Hepatitis C	39 (19.5%)
Alcoholic/Other	36 (18.0%)
Cirrhosis	
Present	127 (63.5%)
Absent	73 (36.5%)
ECOG Performance Status	
0	104 (52.0%)
1	96 (48.0%)
Child-Pugh Class	
A	127 (63.5%)
B	73 (37.5%)
ALBI Grade	
Grade 1	93 (46.5%)
Grade 2	107 (53.5%)
**MELD Score, median (range)**	10 (6–20)
**Neutrophil-Lymphocyte Ratio (NLR), median (range)**	2.6 (1.2–7.3)
**CRP (mg/L), median (range)**	5.0 (0.3–25.0)
**Platelet Count (×10^9^/L), median (range)**	110 (45–220)
**Baseline AFP (ng/mL), median (range)**	200 (5–120,000)
**Liver Stiffness (kPa), median (range)**	16.0 (6.5–40.0)
**Tumor Size (cm), median (range)**	4.8 (2.0–5.5)
Number of Tumor Nodules	
Single	60 (30.0%)
2–3 nodules	120 (60.0%)
>3 nodules	20 (10.0%)
Macrovascular Invasion	
Present	33 (16.5%)
Absent	167 (83.5%)
Ascites at Baseline	
Present	45 (22.5%)
Absent	155 (77.5%)
**TACE Sessions per Patient, mean ± SD (range)**	1.7 ± 0.8 (1–4)
Type of Ablation	
Radiofrequency (RFA)	118 (59.0%)
Microwave (MWA)	82 (41.0%)
**Time Interval (days) Between TACE & PA, median (range)**	10 (7–14)

ECOG, Eastern Cooperative Oncology Group; ALBI, albumin bilirubin; MELD, Model for End-Stage Liver Disease.

In the subgroup analyses, we compared key baseline biomarkers, treatment efficacy, and liver function preservation across age groups (<60 vs. ≥60 years, **Supplementary Table S1**), cirrhosis status (present vs. absent, **Supplementary Table S2**), and etiology (HBV, HCV, alcoholic/other, **Supplementary Table S3**). Younger patients (<60 years) had smaller median tumor sizes (4.5 vs. 5.0 cm, p=0.048), lower AFP levels (150 vs. 320 ng/mL, p=0.032), and lower liver stiffness (15.0 vs. 17.0 kPa, p=0.045) relative to those ≥60 years, with a corresponding improvement in objective response rate (60.2% vs. 48.0%, p=0.041). Among patients with cirrhosis versus those without, AFP was higher in the cirrhotic group (210 vs. 150 ng/mL, p=0.044) and liver stiffness was markedly elevated (20.0 vs. 8.5 kPa, p<0.001). Although cirrhotic versus non-cirrhotic groups showed no significant difference in objective response rate (50.4% vs. 56.2%, p=0.311), cirrhotic patients were significantly less likely to maintain Child-Pugh class A/B (64.3% vs. 82.2%, p=0.007). Regarding etiology, patients with HBV-associated HCC demonstrated higher AFP levels compared to HCV or alcoholic/other etiologies (300 vs. 180 vs. 90 ng/mL, p=0.039), yet no significant differences emerged in tumor size, objective response, or liver function preservation (p>0.05). Collectively, these findings underscore that age, cirrhosis status, and etiology may influence baseline biomarker profiles and, to a lesser extent, treatment outcomes in this HCC population.

### Impact of baseline characteristics on treatment outcomes in HCC patients

In a univariate analysis assessing factors influencing anti-cancer efficacy and liver function in HCC patients treated with TACE and percutaneous ablation, several variables demonstrated significant associations. Patients with baseline AFP levels below 200 ng/mL exhibited higher objective response rates (ORR) of 67.3% and better preservation of Child-Pugh A/B status at 78.2%, compared to those with AFP levels of 200 ng/mL or higher (**Table 2**). Similarly, liver stiffness below 15 kPa was associated with a higher ORR and preservation for liver function (**Table 2**). Patients classified as Child-Pugh Class A showed superior ORR (64.1%) and preservation of liver function (90.6%) than those in Classes B (**Table 2**). Lower neutrophil-to-lymphocyte ratio (NLR) and C-reactive protein (CRP) levels also correlated with improved outcomes, as did smaller tumor sizes (≤5.0 cm) (**Table 2**).

**Table 2:** Univariate analysis of factors associated with anti-cancer efficacy and liver function preservation.

Factor	ORR (%)	p-value	Preserved Child-Pugh (A/B) %	p-value
Baseline AFP (ng/mL)		0.014		0.031
<200	67.3		78.2	
≥200	42.3		57.7	
Liver Stiffness (kPa)		0.027		0.015
<15	65.0		80.0	
≥15	44.7		55.3	
Child-Pugh Class		0.009		<0.001
A	64.1		90.6	
B	37.2		39.5	
NLR (median=2.6)		0.023		0.018
<2.6	65.5		78.2	
≥2.6	46.2		59.6	
CRP (mg/L)		0.041		0.033
<5	63.5		75.0	
≥5	47.7		57.7	
Tumor Size (cm)		0.001		0.002
≤5.0	67.1		80.0	
>5.0	32.4		45.9	

### Multivariate analysis of survival factors in HCC treatment

Next, we performed a multivariate Cox regression analysis for OS in HCC patients treated with TACE and percutaneous ablation. Tumor size remained a robust factor with a HR of 1.88 (95% CI: 1.22–2.90, p=0.004) after adjusting for other variables (**Table 3**). Macrovascular invasion also showed a significant impact with an HR of 1.52 (**Table 3**). Child-Pugh class was another critical determinant, with an HR of 1.94 (**Table 3**). Baseline AFP levels and liver stiffness were associated with HRs of 1.38 (p=0.046) and 1.42 (p=0.050), respectively (**Table 3**). The NLR approached significance, with an HR of 1.24 (p=0.051) (**Table 3**). Factors such as CRP levels and type of ablation did not show a significant impact in the multivariate setting (**Table 3**).

**Table 3:** Multivariate cox regression for overall survival.

Factor	Univariate HR (95% CI)	p-value	Multivariate HR (95% CI)	p-value
**Tumor Size**	1.95 (1.25–3.05)	0.003	1.88 (1.22–2.90)	0.004
**Macrovascular Invasion**	1.60 (1.05–2.44)	0.028	1.52 (1.02–2.26)	0.041
**Child-Pugh Class**	2.10 (1.26–3.51)	0.005	1.94 (1.15–3.27)	0.014
**Baseline AFP**	1.45 (1.04–2.03)	0.029	1.38 (1.01–1.89)	0.046
**Liver Stiffness**	1.52 (1.01–2.28)	0.045	1.42 (1.00–2.02)	0.050
**NLR**	1.30 (1.00–1.70)	0.049	1.24 (1.00–1.54)	0.051
**CRP**	1.20 (0.93–1.55)	0.149		
**Type of Ablation**	1.10 (0.80–1.50)	0.520		

AFP, alpha-fetoprotein; NLR, neutrophil-to-lymphocyte ratio; CRP, Creactive protein.

### Survival analysis by risk level in Kaplan-Meier curves

**Figure 1:**
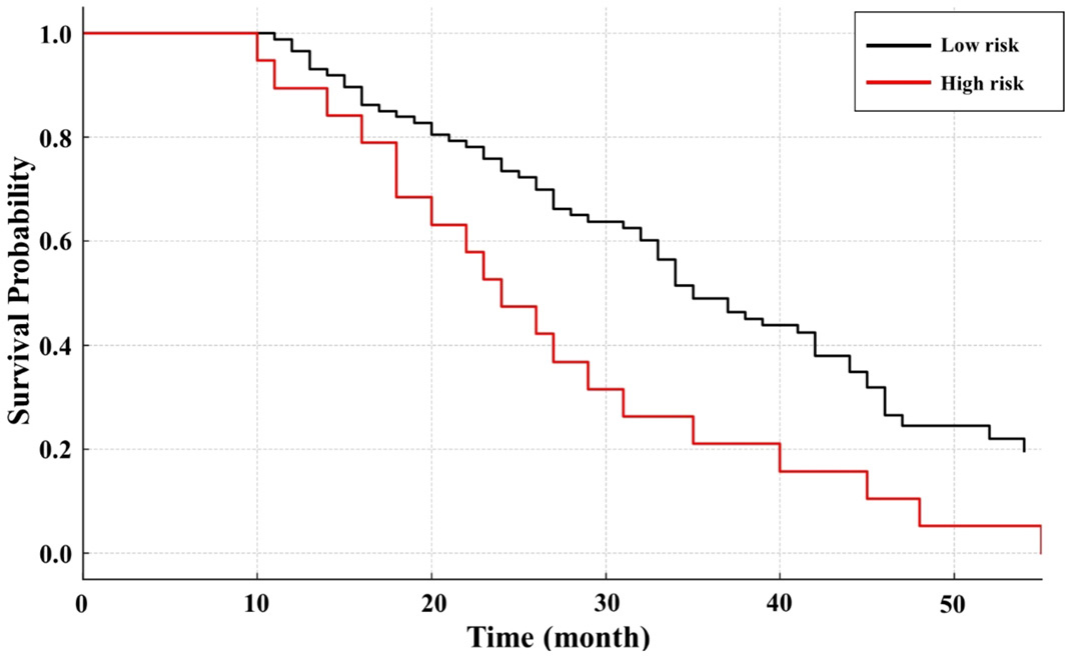
Kaplan-Meier survival curve for HCC patients at high and low risks.

We identified six independent prognostic factors via multivariate Cox regression (including tumor size, Child-Pugh class, and others) and calculated points for each factor. Summing these points yielded a score ranging from 0 (no adverse factors) to a maximum of 13 (all high-weight factors present). We observed that scores from 0 to 6 best captured the low-risk population based on our Kaplan-Meier survival analysis, while patients accumulating more than 6 points had significantly worse outcomes and were thus classified as high-risk (**Figure 1**).

## Discussion

This study explored the efficacy of combining TACE with percutaneous ablation in treating HCC. Our findings underscore the significant impact of this combined treatment approach on enhancing anti-cancer efficacy. Notably, prognostic factors were identified that influenced these outcomes. Factors such as baseline AFP levels, liver stiffness, Child-Pugh class, NLR, and tumor size were significant predictors of treatment success. These findings are critical as they highlight specific patient characteristics and tumor metrics that could be considered in personalizing treatment plans for HCC.

The observed improvement in OS and DFS in our study parallels findings in prior research demonstrating in a meta-analysis that combining TACE with percutaneous ablation significantly improved tumor response and survival rates compared to either treatment alone, particularly in patients with unresectable HCC (19). These improvements in liver function indicators corroborate the findings of Keshavarz et al., who noted enhanced liver function preservation and reduced recurrence rates when TACE was combined with ablation (20). Importantly, our results emphasize the prognostic significance of baseline factors. Patients with lower AFP levels demonstrated superior response rates and better liver function preservation. Similarly, smaller tumor sizes correlated with better outcomes. These findings align with study demonstrating that combining TACE with RFA for small HCC tumors resulted in significantly higher response rates and better survival (21). Lastly, the association of improved outcomes with reduced tumor size and macrovascular invasion aligns with the broader literature on predictive factors in HCC management. Integrated treatment approaches like TACE combined with local therapies offer superior benefits, particularly when addressing high-risk tumor features (22).

Biomarkers play a crucial role in the prognosis and treatment of HCC, a leading cause of cancer-related mortality. Recent studies have identified several key biomarkers, including AFP, glypican 3, and des-gamma-carboxy prothrombin, which are significant for early detection and personalized treatment strategies (23, 24). Advanced technologies such as next-generation sequencing and liquid biopsies have enhanced the understanding of HCC’s molecular landscape, revealing inter- and intra-tumoral heterogeneity that complicates treatment responses (23, 25). Additionally, immunological markers like PD-L1 expression and tumor-infiltrating lymphocytes are critical for predicting immunotherapy efficacy (26). Despite these advancements, challenges remain in biomarker validation and integration into clinical practice, necessitating further research and collaboration to optimize HCC management (23, 26).

Recent studies have identified several promising biomarkers, including CXCL1 and CXCL6, which are upregulated in TACE responders (27). Additionally, traditional biomarkers like alpha-fetoprotein are commonly used, but emerging candidates such as des-gamma-carboxyprothrombin and various microRNAs are being explored for their prognostic capabilities (28, 29). A multi-omics approach is required to better understand tumor characteristics and improve patient stratification for therapies like TACE and ablation (25, 29). Overall, integrating these biomarkers into clinical practice could enhance treatment outcomes and patient management strategies in HCC (30). Our study highlights key differences in patient selection, treatment protocols, and outcome measures that may explain variations in the efficacy of combining TACE with percutaneous ablation for HCC. We found that patients with lower AFP levels and smaller tumors exhibited better response rates and liver function preservation, aligning with findings suggested that the significance of stratifying patients based on liver function and tumor burden (31). AFP is a glycoprotein produced during fetal development, which is re-expressed in HCC, often indicating aggressive disease biology and greater tumor burden (32, 33). TACE reduces blood supply to tumors, inhibiting AFP production, while percutaneous ablation directly destroys AFP-producing cells. Persistently high AFP levels post-procedure may indicate incomplete tumor necrosis or early recurrence (34, 35). Studies have shown that lower baseline AFP correlates with better response to TACE or ablation (35, 36). However, not all HCC patients overexpress AFP, and high AFP levels do not specify lesion location or invasiveness (36). Clinically, AFP serves as a practical indicator for treatment efficacy, with lower levels associated with improved tumor response and survival outcomes (33). However, strict patient selection remains essential to minimize risks and maximize efficacy (37). These findings emphasize the importance of personalized treatment strategies, combining advanced imaging, biomarkers, and multimodal therapies to optimize outcomes in HCC management. Tailoring TACE protocols to patient-specific tumor characteristics could further improves treatment outcomes (38).

While the study provides valuable insights into the efficacy of combining TACE with percutaneous ablation for treating HCC, several limitations must be acknowledged. Firstly, the retrospective nature of the study might introduce potential biases related to data collection and patient selection. Another concern is the generalizability of the findings. The participants, while diverse, may not completely represent all demographics, which can influence treatment responses and outcomes. Furthermore, the study settings and conditions might limit the applicability of the results to settings outside of the study environment, such as different healthcare systems or clinical practices. Addressing these limitations in future research could involve designing prospective studies with broader, more diverse patient populations to enhance the external validity and applicability of the findings.

Our results reinforce the importance of established prognostic factors—such as tumor size and baseline AFP levels—and demonstrate how these can be integrated into a risk-based scoring approach for HCC patients. This scoring model may help refine patient selection and implementation strategies for TACE combined with percutaneous ablation in clinical practice.

## Data Availability

The raw data supporting the conclusions of this article will be made available by the authors, without undue reservation.

## References

[1] Septiarini NA Wardhani P Maimunah U Indrasari YN. Demographics, risk factors, clinical manifestations, staging and liver function tests of hepatocellular Carcinoma: A literature review. World J Of Adv Res Rev. (2024) 24:2087–93. doi: 10.30574/wjarr.2024.24.3.3853

[2] Hwang SY Danpanichkul P Agopian V Mehta N Parikh ND Abou-Alfa GK. Hepatocellular carcinoma: updates on epidemiology, surveillance, diagnosis and treatment. Clin Mol Hepatol. (2024) 31(Suppl):S228–54. doi: 10.3350/cmh.2024.0824 PMC11925437 39722614

[3] Yang S Deng Y Zheng Y Zhang J He D Dai Z. Burden, trends, and predictions of liver cancer in China, Japan, and South Korea: analysis based on the Global Burden of Disease Study 2021. Hepatol Int. (2025). doi: 10.1007/s12072-024-10763-6 PMC12003535 39799268

[4] Patresan J Patel H Chandrasekaran K Reynolds G. Current treatment paradigm and approach to advanced hepatocellular carcinoma. Cureus. (2024) 16:e75471. doi: 10.7759/cureus.75471 39791050 PMC11717138

[5] Danpanichkul P Kim D Pan CW Singal AG Yang JD Wijarnpreecha K. Editorial: steatotic liver diseases emerge as rapidly growing drivers of primary liver cancer in the United States-author’s reply. Aliment Pharmacol Ther. (2025) 61:1059–60. doi: 10.1111/apt.18511 39846176

[6] Wang Q Jia W Liu J Zhao Q Yang Z. Global, regional, and national burden of liver cancer due to alcohol use, 1990-2021: results from the Global Burden of Disease study 2021. Eur J Gastroenterol Hepatol. (2025) 37(4):466–76. doi: 10.1097/MEG.0000000000002899 39621868

[7] Kale SR Karande G Gudur A Garud A Patil MS Patil S. Recent trends in liver cancer: epidemiology, risk factors, and diagnostic techniques. Cureus. (2024) 16:e72239. doi: 10.7759/cureus.72239 39583507 PMC11584332

[8] Teufel A Kudo M Qian Y Daza J Rodriguez I Reissfelder C. Current trends and advancements in the management of hepatocellular carcinoma. Digest Dis (Basel Switzerland). (2024) 42:349–60. doi: 10.1159/000538815 38599204

[9] Haibe Y Kreidieh M El Hajj H Khalifeh I Mukherji D Temraz S. Resistance mechanisms to anti-angiogenic therapies in cancer. Front Oncol. (2020) 10:221. doi: 10.3389/fonc.2020.00221 32175278 PMC7056882

[10] Abdullah SE Perez-Soler R. Mechanisms of resistance to vascular endothelial growth factor blockade. Cancer. (2012) 118:3455–67. doi: 10.1002/cncr.26540 22086782

[11] Ramos P Bentires-Alj M. Mechanism-based cancer therapy: resistance to therapy, therapy for resistance. Oncogene. (2015) 34:3617–26. doi: 10.1038/onc.2014.314 25263438

[12] Sandulache VC Myers JN. Treatment resistance and a glimmer of hope, the promise of new agents. Cambridge, Massachusetts, USA: Academic Press (2020) p. 81–102. doi: 10.1016/B978-0-12-820679-9.00006-2

[13] Kumar A Singh K Kumar K Singh A Tripathi AK Tiwari L. Drug resistance in cancer therapy: mechanisms, challenges and strategies. Asian J Nurs Educ Res. (2024), 30(1):2483094. doi: 10.52711/2349-2996.2024.00019

[14] Lu Y Lu Y. Clinical predictive factors of the efficacy of immune checkpoint inhibitors and kinase inhibitors in advanced hepatocellular cancer. Clin Trans Oncol. (2025) 27(3):1142–54. doi: 10.1007/s12094-024-03644-9 PMC11913906 39158804

[15] Lindner C. Contributing to the prediction of prognosis for treated hepatocellular carcinoma: Imaging aspects that sculpt the future. World J gastrointest Surg. (2024) 16:3377–80. doi: 10.4240/wjgs.v16.i10.3377 PMC11577411 39575286

[16] Gao Y Xu Y Wang Y Lu J Guo JH. Clinical features and prognostic factors of patients with inoperable hepatocellular carcinoma treated with chemotherapy: a population-based study. J gastrointest Oncol. (2024) 15:1122–40. doi: 10.21037/jgo-24-298 PMC11231877 38989427

[17] Huang S Wu D Liao G Liang M Zhang Y Wu H. Identified a novel prognostic model of HCC basing on virus signature for guiding immunotherapy. Discov Oncol. (2024) 15:551. doi: 10.1007/s12672-024-01427-w 39397204 PMC11471745

[18] Das P Spreafico C Sposito C Vaiani M Cascella T Bhoori S. Predictors of good outcomes in patients with hepatocellular carcinoma (HCC) treated with transarterial radioembolization (TARE). J Clin Oncol. (2024) 42:e16219. doi: 10.1200/JCO.2024.42.16_suppl.e16219

[19] Wang W Shi J Xie WF. Transarterial chemoembolization in combination with percutaneous ablation therapy in unresectable hepatocellular carcinoma: a meta-analysis. Liver Int. (2010) 30:741–9. doi: 10.1111/j.1478-3231.2010.02221.x 20331507

[20] Keshavarz P Raman SS. Comparison of combined transarterial chemoembolization and ablations in patients with hepatocellular carcinoma: a systematic review and meta-analysis. Abdomin Radiol (New York). (2022) 47:1009–23. doi: 10.1007/s00261-021-03368-2 34982183

[21] Kim W Cho SK Shin SW Hyun D Lee MW Rhim H. Combination therapy of transarterial chemoembolization (TACE) and radiofrequency ablation (RFA) for small hepatocellular carcinoma: comparison with TACE or RFA monotherapy. Abdomin Radiol (New York). (2019) 44:2283–92. doi: 10.1007/s00261-019-01952-1 30806742

[22] Katsanos K Kitrou P Spiliopoulos S Maroulis I Petsas T Karnabatidis D. Comparative effectiveness of different transarterial embolization therapies alone or in combination with local ablative or adjuvant systemic treatments for unresectable hepatocellular carcinoma: A network meta-analysis of randomized controlled trials. PloS One. (2017) 12:e0184597. doi: 10.1371/journal.pone.0184597 28934265 PMC5608206

[23] Yu B Ma W. Biomarker discovery in hepatocellular carcinoma (HCC) for personalized treatment and enhanced prognosis. Cytokine Growth factor Rev. (2024) 79:29–38. doi: 10.1016/j.cytogfr.2024.08.006 39191624

[24] Attia AM Rezaee-Zavareh MS Hwang SY Kim N Adetyan H Yalda T. Novel biomarkers for early detection of hepatocellular carcinoma. Diagn (Basel Switzerland). (2024) 14:2278. doi: 10.3390/diagnostics14202278 PMC11507329 39451600

[25] Chan YT Zhang C Wu J Lu P Xu L Yuan H. Biomarkers for diagnosis and therapeutic options in hepatocellular carcinoma. Mol Cancer. (2024) 23(1):189. doi: 10.1186/s12943-024-02101-z 39242496 PMC11378508

[26] Taherifard E Tran K Saeed A Yasin JA Saeed A. Biomarkers for immunotherapy efficacy in advanced hepatocellular carcinoma: A comprehensive review. Diagn (Basel Switzerland). (2024) 14:2054. doi: 10.3390/diagnostics14182054 PMC11431712 39335733

[27] Kinzler MN Bankov K Bein J Döring C Schulze F Reis H. CXCL1 and CXCL6 are potential predictors for HCC response to TACE. Curr Oncol (Toronto Ont.). (2023) 30:3516–28. doi: 10.3390/curroncol30030267 PMC10046993 36975480

[28] Zavadil J Rohan T Juráček J Kiss I Ostřížková L Válek V. Biomarkers as prognostic and predictive factors in patients with hepatocellular carcinoma undergoing radiological oncological interventions. Biomarkery jako prognostické a prediktivní faktory u pacientů s hepatocelulárním karcinomem podstupujících radiologické onkologické intervence. Klinicka onkol: casopis Ceske Slovenske onkol spolecnosti. (2023) 36:104–11. doi: 10.48095/ccko2023104 37072244

[29] Casadei-Gardini A Orsi G Caputo F Ercolani G. Developments in predictive biomarkers for hepatocellular carcinoma therapy. Expert Rev Anticancer Ther. (2020) 20:63–74. doi: 10.1080/14737140.2020.1712198 31910040

[30] Meng M Li W Yang X Huang G Wei Z Ni Y. Transarterial chemoembolization, ablation, tyrosine kinase inhibitors, and immunotherapy (TATI): A novel treatment for patients with advanced hepatocellular carcinoma. J Cancer Res Ther. (2020) 16:327–34. doi: 10.4103/jcrt.JCRT_101_20 32474520

[31] Galun D Basaric D Zuvela M Bulajic P Bogdanovic A Bidzic N. Hepatocellular carcinoma: From clinical practice to evidence-based treatment protocols. World J Hepatol. (2015) 7:2274–91. doi: 10.4254/wjh.v7.i20.2274 PMC4568488 26380652

[32] Ramakrishnan K Sanjeev D Rehman N Raju R. A network map of intracellular alpha-fetoprotein signalling in hepatocellular carcinoma. J Viral hepatitis. (2025) 32:e14035. doi: 10.1111/jvh.14035 39668590

[33] Yeo YH Lee YT Tseng HR Zhu Y You S Agopian VG. Alpha-fetoprotein: Past, present, and future. Hepatol Commun. (2024) 8:e0422. doi: 10.1097/HC9.0000000000000422 38619448 PMC11019827

[34] Haque M Yasmin MR. Role of serum alpha-fetoprotein in assessing the resectability of hepatocellular carcinoma. SAS J Surg. (2024) 10:1189–98. doi: 10.36347/sasjs.2024.v10i10.020

[35] Al-Hasan M Mehta N Yang JD Singal AG. Role of biomarkers in the diagnosis and management of HCC. Liver Transplant. (2024). doi: 10.1097/LVT.0000000000000398 38738964

[36] Fadillah FR Rotty LWA Sugeng C. Hubungan kadar alpha fetoprotein dengan derajat keparahan karsinoma hepatoseluler. Med Scope J (MSJ). (2024) 7:74–9. doi: 10.35790/msj.v7i1.54829

[37] Livraghi T. Radiofrequency ablation, PEIT, and TACE for hepatocellular carcinoma. J hepato-biliary-pancreatic Surg. (2003) 10:67–76. doi: 10.1007/s10534-002-0714-y 12918460

[38] Esagian SM Kakos CD Giorgakis E Burdine L Barreto JC Mavros MN. Adjuvant transarterial chemoembolization following curative-intent hepatectomy versus hepatectomy alone for hepatocellular carcinoma: A systematic review and meta-analysis of randomized controlled trials. Cancers. (2021) 13:2984. doi: 10.3390/cancers13122984 34203692 PMC8232114

