# Supplementary material for: Longitudinal study of factors associated with the anti-cancer efficacy and liver function in HCC patients treated with TACE in combination with percutaneous ablation
Source: Front Oncol. 2025 Apr 16;15:1566865. doi: 10.3389/fonc.2025.1566865 (PMC12040659; doi:10.3389/fonc.2025.1566865)
Supplement: Supplementary file 1 [file Table1.docx]

## **Supplementary Table S1. Baseline Biomarkers by Age Group**

|  | **<60 Years old**  **(n = 98)** | **≥60 Years old**  **(n = 102)** | **p-value** |
| --- | --- | --- | --- |
| **Tumor Size (cm)** | 4.5 (2.2–5.2) | 5.0 (2.0–5.5) | 0.048 |
| **AFP (ng/mL)** | 150 (10–120,000) | 320 (5–110,000) | 0.032 |
| **Liver Stiffness (kPa)** | 15.0 (7.0–38.0) | 17.0 (6.5–40.0) | 0.045 |
| **NLR** | 2.5 (1.2–7.3) | 2.7 (1.3–7.1) | 0.085 |
| **CRP (mg/L)** | 4.3 (0.3–25.0) | 5.5 (0.4–24.0) | 0.074 |
| **Objective Response Rate** | 60.2% | 48.0% | 0.041 |
| **Preserved Child-Pugh (A/B)** | 80.6% | 68.6% | 0.037 |

Note: AFP: alpha-fetoprotein; NLR: neutrophil-to-lymphocyte ratio; CRP: C-reactive protein
