# Supplementary material for: Longitudinal study of factors associated with the anti-cancer efficacy and liver function in HCC patients treated with TACE in combination with percutaneous ablation
Source: Front Oncol. 2025 Apr 16;15:1566865. doi: 10.3389/fonc.2025.1566865 (PMC12040659; doi:10.3389/fonc.2025.1566865)
Supplement: Supplementary file 2 [file Table2.docx]

## **Supplementary Table S2. Baseline Biomarkers by Cirrhosis Status**

|  | **Cirrhosis**  **(n = 127)** | **No Cirrhosis**  **(n = 73)** | **p-value** |
| --- | --- | --- | --- |
| **Tumor Size (cm)** | 4.9 (2.0–5.5) | 4.5 (2.0–5.5) | 0.110 |
| **AFP (ng/mL)** | 210 (5–120,000) | 150 (5–110,000) | 0.044 |
| **Liver Stiffness (kPa)** | 20.0 (10.0–40.0) | 8.5 (6.5–17.0) | <0.001 |
| **NLR** | 2.8 (1.3–7.3) | 2.3 (1.2–5.2) | 0.052 |
| **CRP (mg/L)** | 5.8 (0.3–25.0) | 4.1 (0.3–16.0) | 0.091 |
| **Objective Response Rate** | 50.4% | 56.2% | 0.311 |
| **Preserved Child-Pugh (A/B)** | 64.3% | 82.2% | 0.007 |

Note: AFP: alpha-fetoprotein; NLR: neutrophil-to-lymphocyte ratio; CRP: C-reactive protein
