# Supplementary material for: Longitudinal study of factors associated with the anti-cancer efficacy and liver function in HCC patients treated with TACE in combination with percutaneous ablation
Source: Front Oncol. 2025 Apr 16;15:1566865. doi: 10.3389/fonc.2025.1566865 (PMC12040659; doi:10.3389/fonc.2025.1566865)
Supplement: Supplementary file 3 [file Table3.docx]

**Supplementary Table S3. Baseline Biomarkers by Etiology**

| **Parameter** | **HBV**  **(n = 125)** | **HCV**  **(n = 39)** | **Alcoholic/Other**  **(n = 36)** | **p-value** |
| --- | --- | --- | --- | --- |
| **Tumor Size (cm)** | 4.9 (2.0–5.5) | 4.5 (2.3–5.3) | 4.6 (2.0–5.3) | 0.179 |
| **AFP (ng/mL)** | 300 (10–120,000) | 180 (5–50,000) | 90 (5–80,000) | 0.039 |
| **Liver Stiffness (kPa)** | 17.0 (7.0–40.0) | 15.0 (7.0–35.0) | 15.0 (6.5–28.0) | 0.085 |
| **NLR** | 2.7 (1.2–7.3) | 2.5 (1.2–6.0) | 2.4 (1.3–6.5) | 0.211 |
| **CRP (mg/L)** | 5.2 (0.3–25.0) | 4.8 (1.0–22.0) | 4.0 (0.3–24.0) | 0.322 |
| **Objective Response Rate** | 54.4% | 60.0% | 58.3% | 0.499 |
| **Preserved Child-Pugh (A/B)** | 68.8% | 74.4% | 72.2% | 0.603 |

Note: AFP: alpha-fetoprotein; NLR: neutrophil-to-lymphocyte ratio; CRP: C-reactive protein
